# Serum Autotaxin is a Marker of the Severity of Liver Injury and Overall Survival in Patients with Cholestatic Liver Diseases

**DOI:** 10.1038/srep30847

**Published:** 2016-08-10

**Authors:** Ewa Wunsch, Marcin Krawczyk, Malgorzata Milkiewicz, Jocelyn Trottier, Olivier Barbier, Markus F. Neurath, Frank Lammert, Andreas E. Kremer, Piotr Milkiewicz

**Affiliations:** 1 Department of Clinical and Molecular Biochemistry, Pomeranian Medical University in Szczecin, 70-111 Szczecin, Poland; 2 Department of Medicine II, Saarland University Medical Center, Saarland University, 66421 Homburg, Germany; 3 Laboratory of Metabolic Liver Diseases, Department of General, Transplant and Liver Surgery, Medical University of Warsaw, 02-091 Warsaw, Poland; 4 Department of Medical Biology, Pomeranian Medical University in Szczecin, 70-111 Szczecin, Poland; 5 Laboratory of Molecular Pharmacology, CHU-de-Québec & Faculty of Pharmacy, Laval University, 2705 Québec, QC, Canada; 6 Department of Medicine I, Friedrich-Alexander-University Erlangen-Nürnberg, 91054 Erlangen, Germany; 7 Liver and Internal Medicine Unit, Department of General, Transplant and Liver Surgery of the Medical University of Warsaw, 02-097 Warsaw, Poland

## Abstract

Autotaxin (ATX) is involved in the synthesis of lysophosphatidic acid. Both have recently been linked to cholestatic pruritus and liver injury. We aimed to investigate whether ATX is an indicator of cholestatic liver injury, health-related quality of life (HRQoL) and prognosis based on a group of 233 patients, 118 with primary biliary cholangitis (PBC) and 115 with primary sclerosing cholangitis (PSC). Patients were followed for 1–60 months, cumulative survival rates were calculated. ATX activity was significantly higher in both groups than in the 103 controls, particularly in patients with cirrhosis and in patients with longer disease duration. Ursodeoxycholic acid (UDCA) non-responders with PBC exhibited increased ATX activity. ATX activity was correlated with liver biochemistry, MELD, Mayo Risk scores and was associated with worse disease-specific HRQoL aspects. In both groups, Cox model analysis indicated that ATX was a negative predictor of survival. Increased ATX levels were associated with a 4-fold higher risk of death/liver transplantation in patients with PBC and a 2.6-fold higher risk in patients with PSC. We conclude that in patients with cholestatic conditions, ATX is not only associated with pruritus but also indicates impairment of other HRQoL aspects, liver dysfunction, and can serve as a predictor of survival.

Pruritus is a frequent symptom in chronic cholestatic liver conditions such as primary biliary cholangitis (PBC) and primary sclerosing cholangitis (PSC). Recent studies have identified lysophosphatidic acid (LPA) as a potential mediator of pruritus associated with cholestasis^1^. LPA is a small but powerful signalling molecule that acts through at least 6 different G protein-coupled receptors in many cell types with a variety of actions^2^. Because LPA in plasma and serum is unstable with increasing concentrations during storage^3^, the activity and protein content of autotaxin (ATX) in serum are considered a valid indicator of LPA levels. ATX is a secreted enzyme with lysophospholipase D activity and hydrolyses lysophosphatidylcholine (LPC) into LPA. ATX is considered a main source of extracellular LPA^4^. Serum ATX activity has recently been shown to be increased in patients with pruritus due to cholestasis^1^. Moreover, ATX levels were closely correlated with itch intensity and decreased with the successful treatment of pruritus in cholestatic patients^5^. These observations, establishing ATX as a serum marker of cholestatic itch, were further strengthened by our recent genetic analysis identifying a common variant in the LPA metabolism pathway that might protect against cholestatic pruritus^6^.

Patients with chronic liver disease suffer not only from pruritus but also from numerous other debilitating symptoms, such as fatigue and depression. A significant proportion of patients with chronic cholestasis report a low quality of life^7^. However, to date, the mechanisms underlying these symptoms in the setting of chronic cholestasis have not been elucidated. Given the above-mentioned association between ATX/LPA and cholestatic itch, we hypothesized that these molecules could affect domains of quality of life other than pruritus. To this end, we analysed two large cohorts of prospectively recruited patients with PBC and PSC. A thorough clinical work-up was performed, and serum ATX was measured (both activity and protein concentration) in all patients. Subsequently, we analyzed serum ATX levels in relation to the results of the symptom assessment tests and cholestatic markers as well as serum bile acid concentrations. Additionally, in view of recent findings linking ATX levels to the severity of liver disease and overall survival in cirrhotic patients^8^, we examined the potential relationship between ATX and markers of liver injury, prognostic indicators and survival data. These analyses demonstrated, for the first time, that increased serum ATX activity and protein levels are associated with several aspects of quality of life in cholestatic patients as well as with markers of cholestatic liver injury and higher risks of death and transplantation.

## Results

### Patients with cholestasis exhibit increased serum ATX levels

**Figure 1:**
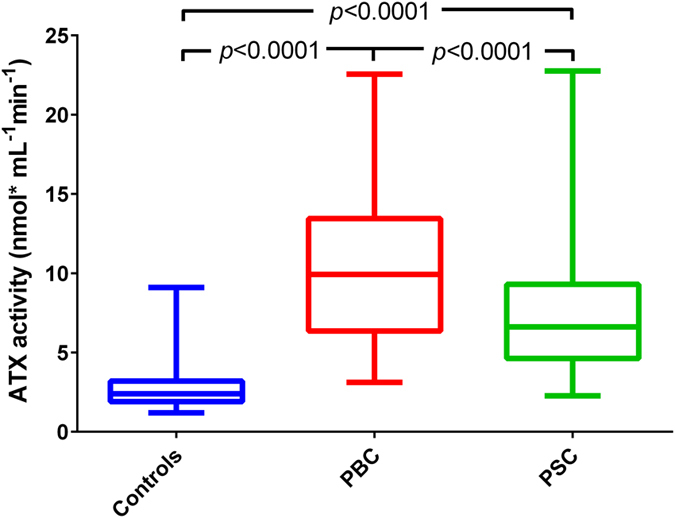
ATX activity in both groups compared to healthy controls. ANOVA analysis. The data are presented in the box-and-whisker plot with median values indicated by the middle line.

PBC and PSC patients had increased ATX activity compared to healthy controls (10.2 ± 4.4 *vs*. 7.3 ± 3.4 *vs*. 2.8 ± 1.4 nmol mL^−1^ min^−1^ in patients with PBC, PSC and controls, respectively; *P* < 0.0001 for all; Fig. 1). An analysis of all patients revealed that the mean ATX activity of patients in the PBC group was significantly higher than in patients in the PSC group (10.2 ± 4.4 *vs*. 7.3 ± 3.4 nmol mL^−1^ min^−1^, *P* < 0.0001; Fig. 1). This significant difference between groups was also present when taking into account only patients with liver cirrhosis (11.7 ± 3.5 *vs*. 9.3 ± 3.8 nmol mL^−1^ min^−1^, *P* < 0.01) or patients without liver cirrhosis (9.1 ± 4.7 *vs*. 6.6 ± 3.0 nmol mL^−1^ min^−1^, *P* < 0.001, in PBC and in PSC, respectively). In healthy controls, ATX activity was higher in females (3.1 ± 1.7 *vs*. 2.5 ± 0.7 nmol mL^−1^ min^−1^ in males, *P = *0.01) and was associated with age (*r = *0.31, *P = *0.012). In both cholestatic groups analysed separately, ATX activity was independent from age and gender, even if cirrhotic patients were excluded (Supplementary Figure 1). However, when we analysed both cholestatic groups together, ATX activity was enhanced in females (9.6 ± 4.4 *vs*. 7.3 ± 3.5 nmol mL^−1^ min^−1^ in males) and was dependent on patient age (r = 0.258, *P* < 0.0001). ATX activity was strongly correlated with ATX protein levels (Spearman’s Rho = 0.93, *P* < 0.0001; Supplementary Figure 2).

### Serum autotaxin levels are associated with liver disease severity

**Figure 2:**
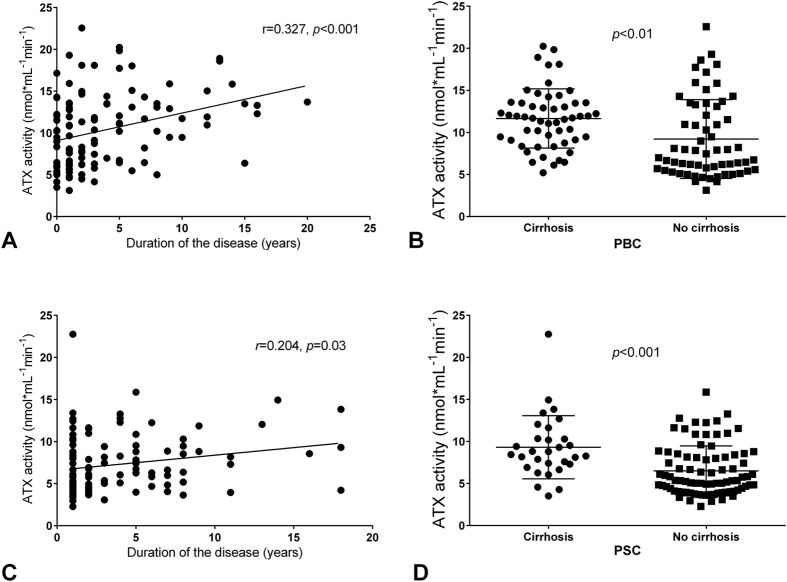
The relationship between ATX activity and the presence of cirrhosis and disease duration. Pearson’s correlation coefficients between ATX activity and the duration of the liver disease in patients with (**A**) PBC and (**B**) PSC. Comparisons between ATX activity in cirrhotic and non-cirrhotic patients with (**C**) PBC and (**D**) PSC. ANOVA. The data are presented as mean values ± SD.

In the PBC cohort, ATX activity was higher in patients with a longer duration of the disease (r = 0.327, *P* < 0.001; Fig. 2A) and patients with liver cirrhosis (11.6 ± 3.5 *vs*. 9.1 ± 4.7 nmol mL^−1^ min^−1^ in non-cirrhotic stage, *P* < 0.01; Fig. 2B). Although there were no associations between Child-Pugh classes and ATX levels in cirrhotic patients (11.5 ± 3.5 *vs*. 12.1 ± 4.1 *vs*. 11.0 ± 2.0 nmol mL^−1^ min^−1^ in Child-Pugh stage A, B and C, respectively; *P* > 0.05 for all; Supplementary Figure 3A), the AUC analysis identified serum ATX activity as a significant predictor of cirrhosis (AUC = 0.7; 95%CI = 0.60–0.79; *P* < 0.001; Supplementary Figure 4A).

**Figure 3:**
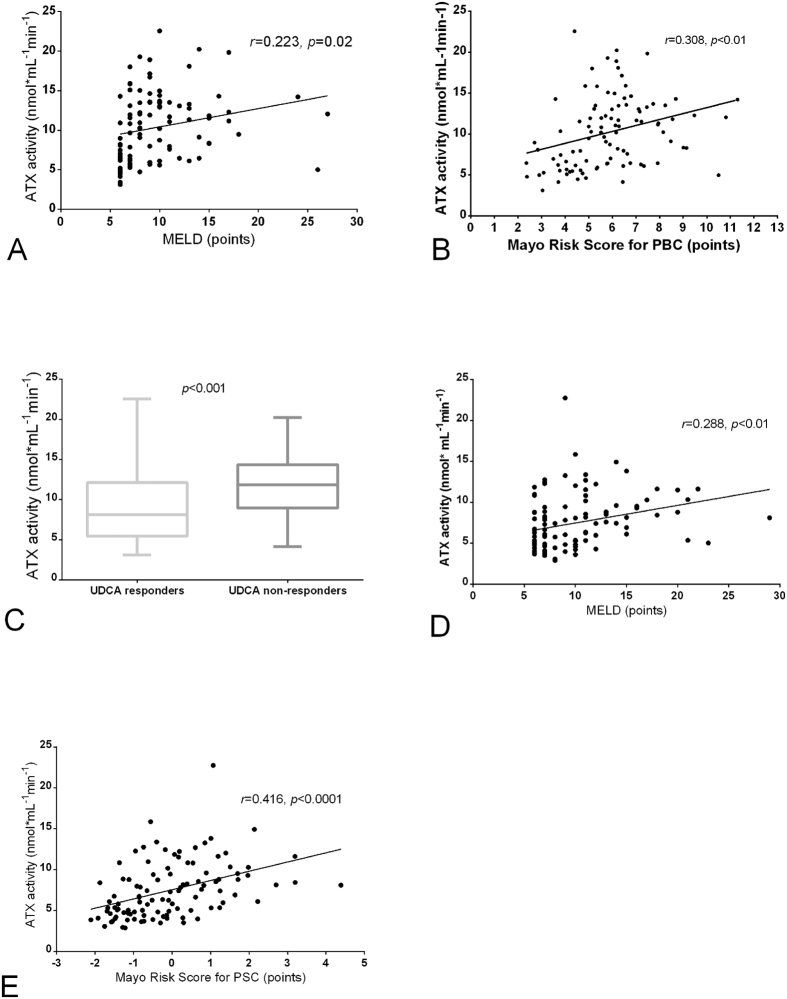
Correlations among ATX activity, UDCA response, and MELD and Mayo Risk scores for patients with PBC and PSC. Pearson’s correlation coefficients of ATX activity and MELD score in patients with (**A**) PBC and (**D**) PSC. (**B**) Mayo Risk score for PBC and (**E**) Mayo Risk score for PSC. (**C**) Comparison of ATX activity between UDCA responders and non-responders in the PBC group. ANOVA. The data are presented in the box-and-whisker plot with median values indicated by the middle line.

In addition, ATX levels were significantly correlated (all *P* < 0.05) with several laboratory markers of disease severity such as ALP, albumin and haemoglobin (Table 1) as well as with the MELD score (r = 0.223, *P = *0.02; Fig. 3A). In the multivariate linear regression model, ALP and haemoglobin levels were independently associated with serum ATX (*P* < 0.0002). We also detected a significant correlation between ATX activity and the Mayo Risk score in patients with PBC (r = 0.308, *P* < 0.01; Fig. 3B), which was also an independent variable in the multivariate analysis (*P* < 0.0002). These correlations remained statistically significant when cirrhotic and non-cirrhotic patients were analysed separately, particularly in non-cirrhotic patients (Supplementary Table 1A). Finally, ursodeoxycholic acid (UDCA) non-responders exhibited significantly higher ATX activity than the patients who responded to UDCA (11.9 ± 4.2 *vs*. 9.1 ± 4.2 nmol mL^−1^ min^−1^, *P* < 0.001; Fig. 3C).

Likewise, in the PSC group, ATX activity was associated with disease duration (r = 0.204, *P = *0.03; Fig. 2C) and was significantly higher in cirrhotic patients than in subjects without cirrhosis (9.3 ± 3.8 *vs*. 6.6 ± 3.0 nmol mL^−1^ min^−1^, *P* < 0.001; Fig. 2D). There were, however, no differences in ATX levels among cirrhotic patients in different Child-Pugh classes (9.0 ± 4.7 *vs*. 9.8 ± 2.5 *vs*. 9.2 ± 1.5 nmol mL^−1^ min^−1^ in Child-Pugh classes A, B and C, respectively; *P* > 0.05 for all; Supplementary Figure 3B). The AUC analysis identified serum ATX activity as a significant predictor of cirrhosis in patients with PSC (AUC = 0.734; 95%CI = 0.63–0.83, *P* < 0.001; Supplementary Figure 4B). In the univariate analysis, ATX activity was correlated with ALP and AST concentrations as well as with levels of albumin, INR values, platelet counts and haemoglobin levels (Table 1). In the multivariate linear regression model of ALP, haemoglobin levels and platelet counts were identified as independent variables (*P* < 0.0001). Significant associations were detected between ATX activity and MELD score (r = 0.288, *P* < 0.01; Fig. 3D) and between ATX activity and Mayo Risk score in patients with PSC (r = 0.416, *P* < 0.0001; Fig. 3E). The Mayo Risk score was also independently correlated with ATX levels in the multivariate analysis (*P* < 0.0001). These correlations were also present in non-cirrhotic patients when cirrhotic and non-cirrhotic patients were analysed separately (Supplementary Table 1B).

### Serum autotaxin levels are correlated with bile acid concentrations and itch intensity

As demonstrated in Table 2, ATX activity in patients with PBC was associated with total serum bile acids concentration (r = 0.412, *P* < 0.0001). With respect to single bile acids species in patients with PBC, the strongest correlations in the univariate analysis were obtained for glycine and taurine derivatives of the primary acids chenodeoxycholic acid (CDCA) and cholic acid (CA) (Table 2). In the multivariate linear regression model, glycine species of CA and taurine derivatives of lithocholic acid were independently associated with serum ATX activity (*P* < 0.0001). Moreover, ATX activity was significantly associated with pruritus intensity as measured by the *Itch* domain of the PBC-40 and PBC-27 questionnaires (r = 0.305, *P* < 0.01; Table 3). This correlation remained when cirrhotic patients were excluded from the analysis (r = 0.290, *P = *0.02).

In accordance with these findings in the PBC cohort, we detected significant correlations between ATX activity and total bile acids levels (r = 0.409, *P* < 0.0001) and various bile acids derivatives (Table 2) in the PSC patients as well. In the multivariate linear regression analysis, glycine species of CDCA was significantly associated with serum ATX concentration (*P* < 0.0001). The correlation between the *Itch* domain and ATX concentrations was even stronger than in patients in the PBC group (r = 0.376, *P* < 0.0001; Table 3), and this correlation remained significant when cirrhotic patients were excluded from the analysis (r = 0.315, *P* < 0.01).

Total bile acids concentration was markedly elevated in patients from both groups compared to healthy control patients (3110.0 ± 2264.5 nM in controls *vs*. 43 850.1 ± 47081.1 nM in patients with PBC, *P* < 0.0001; 3110.0 ± 2264.5 nM in controls *vs*. 44 751.7 ± 58 058.1 nM in patients with PSC, *P* < 0.0001). In contrast to findings in both groups, there was no correlation between ATX activity and total serum bile acids level in healthy controls (r = 0.013, *P* = 0.89).

### Serum autotaxin levels are indicators of HRQoL in patients with cholestatic liver disease

The results of the quality-of-life questionnaires in both groups are presented in Supplementary Table 2. As shown in Table 3, the activity of ATX was correlated with several disease-specific aspects of quality of life in patients with PBC: the *Fatigue* and *Cognitive* domains of both the PBC-40 and PBC-27 questionnaires and the *Emotional* domain in the PBC-27 questionnaire. Among these aspects, scores in the *Fatigue* domain were correlated independently with ATX activity in the multivariate regression model (*P* < 0.04 on the PBC-40 and *P* < 0.03 on the PBC-27 questionnaire). ATX activity was associated with scores in the *Physical Functioning* domain on the generic SF-36 questionnaire. In patients in the PSC group, no associations were detected between ATX activity and HRQoL measures in either the PBC-40/PBC-27 or SF-36 questionnaires, with the exception of the *Itch* domain.

### Autotaxin is a negative predictor of survival in patients with cholestatic liver diseases

**Figure 4:**
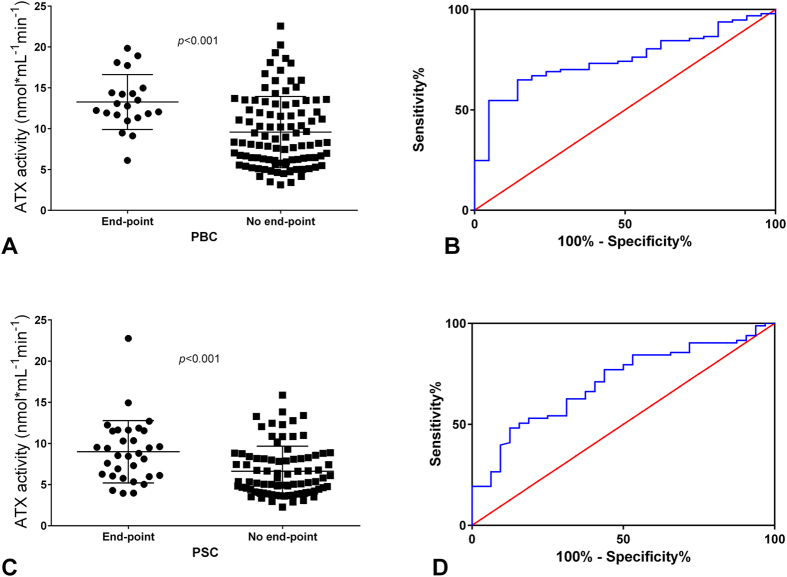
The relationship between survival and ATX activity. A comparison of ATX activity between patients who reached an end-point during the follow-up (underwent liver transplantation or died) and patients who were alive at the end of the study in the (**A**) PBC and (**C**) PSC groups. Receiver operating characteristic (ROC) area under the curve (AUC) in patients with (**B**) PBC and (**D**) PSC.

Patients with PBC were followed up for 1–58 months (mean follow-up time: 11.5 ± 9.2 months). During this time, a total of 21 (17.8%) patients reached end-points: 18 (15.3%) patients were transplanted, and 3 (2.5%) died. Among them, 17 patients had cirrhosis. The event-free survival rate in the PBC group was 82.2%. The cumulative survival probability is presented in Supplementary Figure 5A. In PBC patients who reached an end-point in the follow-up period (liver transplantation or death), the activity of ATX was significantly higher than in the remaining patients (13.3 ± 3.4 *vs*. 9.6 ± 4.4 nmol mL^−1^ min^−1^, *P* < 0.001; Fig. 4A). Overall, ATX activity was a negative survival predictor in this group (chi^2^ = 6.749, *P* < 0.01). The receiver operating characteristic (ROC) area under the curve (AUC) indicated that the optimal cut-off of ATX activity for discriminating patients with a higher risk of death/liver transplantation was 11.5 nmol mL^−1^ min^−1^, with a sensitivity of 76.2% and a specificity of 68.0% (Fig. 4B). The Cox regression model for increased ATX values revealed a hazard ratio (HR) of 3.87 (*P* < 0.01, 95%CI = 1.4 –10.7). To investigate whether ATX is an independent predictor of survival, we performed multivariate survival analysis using the Cox regression model. In the first step, we indicated other variables influencing survival in the univariate analysis: UDCA non-response status (chi^2^ = 8.617, *P* = 0.003), presence of cirrhosis (chi^2^ = 7.417 *P* = 0.007), Mayo Risk score for PBC (chi^2^ = 8.725, *P* = 0.003) and MELD score (chi^2^ = 8,168, *P* = 0.004). Among these factors, ATX, MELD score and UDCA non-response were the independent variables according to multivariate analysis (Supplementary Table 3A).

Patients with PSC were followed up for 1–60 months (mean follow-up time: 12.6 ± 11.8 months). During the follow-up period, a total of 32 (27.8%) patients reached end-points: 28 (24.3%) had liver transplantation, and 4 died (3.5%). Among them, 17 patients had cirrhosis. The event-free survival rate in the PSC group was 72.2%. The cumulative survival probability of this group is presented in Supplementary Figure 5B. ATX activity was significantly higher in patients who reached end-points (liver transplantation or death) in comparison to those who were alive or not transplanted during the follow-up period (9.0 ± 3.9 *vs*. 6.6 ± 3.0 nmol mL^−1^ min^−1^, *P* < 0.001; Fig. 4C). In the univariate Cox regression model, ATX concentration was significantly associated with overall survival (chi^2^ = 6.73, *P* < 0.01). Receiver operating characteristic (ROC) area under the curve (AUC) values indicated that the optimal cut-off of ATX activity with which to distinguish patients with a higher risk of end-points was 7.5 nmol mL^−1^ min^−1^, with a sensitivity of 62.5% and a specificity of 66.3% (Fig. 4D). With this cut-off level, the Cox regression model demonstrated an HR of 2.63 (*P* < 0.01, 95%CI = 1.3–5.4). To investigate whether ATX is an independent predictor of survival, we performed a multivariate survival analysis using the Cox regression model. Firstly, we identified other variables influencing survival in the univariate analysis: the presence of cirrhosis (chi^2^ = 5.4, *P* = 0.02) and MELD score (chi^2^ = 32.562, *P* < 0.0001). Among the included variables, multivariate survival analysis using Cox regression revealed the MELD score as the only independent variable in patients with PSC (Supplementary Table 3B).

## Discussion

The ATX-LPA-axis has been implicated in the occurrence of pruritus during cholestasis and in the presence of liver fibrosis and cirrhosis. This study adds several novel findings of elevated clinical and therapeutic relevance. Our results showed that in chronic cholestasis, ATX activity is strongly correlated not only with itch intensity but also with the impairment of other disease-specific aspects of patients’ quality of life. Interestingly, ATX levels were also associated with the severity of liver disease. In both cohorts, ATX levels were correlated with ALP as well as MELD and Mayo Risk scores. Moreover, ATX levels were higher in patients with liver cirrhosis and a longer duration of the disease. Amongst patients with PBC, UDCA non-response was associated with higher ATX activity. Finally, our study indicated that ATX was a potential predictor of overall survival in patients with cholestatic conditions.

Recent studies have described a potential role of the ATX-LPA-axis in the pathogenesis of cholestatic itch. The small molecule LPA was identified as a potent neuronal activator in the sera of cholestatic patients suffering from pruritus^1^. Subsequently, ATX activity was markedly elevated in chronic cholestasis and a strong elevation was only observed in patients with cholestatic pruritus but not other forms of pruritus^5^. Although elevated ATX has been demonstrated in other conditions with concomitant itch such as atopic dermatitis, uraemia and Hodgkin lymphoma^9^,^10^,^11^, ATX activity was directly correlated with the intensity of pruritus only in cholestasis and atopic dermatitis. Furthermore, successful treatment of pruritus was associated with a reduction in ATX serum activity in all analysed therapeutic options^5^. Moreover, in intrahepatic cholestasis during pregnancy, ATX activity was increased compared to other pregnancy related disorders and to ATX activity in healthy pregnant women^12^. Finally, in a recent study, serum ATX activity was markedly elevated and correlated with itch intensity in children with cholestatic diseases^13^.

The assessment of the relationship between itch intensity and ATX was previously based on the visual analogue scale and data analysing other disease-related symptoms were lacking. In our study, we analysed in detail the association between ATX and pruritus as well as the links between ATX and other aspects of HRQoL in large, well-defined cohorts of patients with PBC and PSC. We used, for the first time in this context, both the generic questionnaire SF-36, which estimates general patient well-being, as well as the disease-specific assessments PBC-40 and PBC-27. PBC-40 and PBC-27 were designed for the assessment of symptoms more specifically related to PBC, which include pruritus, chronic fatigue and cognitive and emotional decline^14^, and recently, we demonstrated the usefulness of these questionnaires in the evaluation of the quality of life in patients with PSC^15^,^16^. In the present study, ATX was significantly associated with scores in the *Itch* domain of the PBC-40/PBC-27 in both cohorts. This finding confirms the pruritogenic role of LPA in patients with chronic cholestatic conditions. Of interest, ATX activity was also correlated with other disease-specific aspects of HRQoL in the PBC group, namely, *Fatigue*, *Cognitive* and *Emotional* domains; however, no relationship between enzymatic activity and ATX protein levels (except for the *Physical Functioning* domain) was seen for the generic SF-36 questionnaire. Notably, in our previous studies on HRQoL in patients with chronic cholestasis, there were only a few associations between laboratory parameters and quality-of-life aspects in addition to a strong link between ALP and *Itch* domains^15^,^17^. This finding supports the notion that ATX, unlike other laboratory parameters, is closely associated with disease-specific symptoms in PBC patients and that ATX inhibitors or LPA receptor antagonists may be of potential benefit in the treatment of pruritus and other symptoms related to chronic cholestasis.

In our study, serum ATX was not only associated with indices of patient well-being but was also associated with several laboratory parameters. We noted significant correlations with ALP levels in both groups and with AST levels in the PSC cohort. Higher levels of ATX were correlated with worse liver function (lower albumin levels in both groups and higher INR values in PSC patients) and lower platelet counts in PSC and haemoglobin concentrations in both cohorts. Moreover, ATX activity was significantly correlated with MELD scores in both groups. Interestingly, when we analysed non-cirrhotic and cirrhotic patients separately, most associations remained significant in patients in the early stage of liver disease, but not in patients with cirrhosis. This observation suggests that ATX is a valuable indicator of liver injury even in patients without cirrhosis. However, enzyme activity was also higher in patients with cirrhosis and with a longer duration of the disease in both cohorts. Finally, AUC analysis identified serum ATX as a significant indicator of cirrhosis in both PSC and PBC patients. The only exception was a lack of statistical significance with respect to Child-Pugh scores calculated for cirrhotic patients, which may be due to the small number of subjects in each class.

The correlation between ATX levels and the severity of liver disease has been recently indicated by Pleli *et al*., who reported that in patients with liver cirrhosis, serum ATX levels were increased compared to healthy subjects and ATX levels were correlated with Child-Pugh and MELD scores. Furthermore, lower ATX levels were independently associated with a longer overall survival in patients^8^. Notably, in the aforementioned study, the aetiology of liver disease was primarily viral or alcoholic. Our study focuses on chronic cholestatic conditions, and in addition to general laboratory and clinical parameters, we investigated cholestasis specific issues such as cholestasis-related symptoms and prognostic scales. We have shown that ATX levels are independently correlated with both the Mayo Risk score for PBC and the Mayo Risk score for PSC. Finally, we indicated that UDCA non-response was associated with a marked increase of serum ATX activity in PBC patients. This is a noteworthy observation because UDCA non-response is considered a negative prognostic factor in this condition^18^. However, this association is most likely due to the presence of more severe cases of liver disease in this group of patients. These findings indicate that ATX can potentially serve as an indicator of the severity of liver disease, even in non-cirrhotic patients, and may represent a novel marker of UDCA non-response. In addition to these results, ATX activity was a significant predictor of death and liver transplantation in both cholestatic cohorts, and in the multivariate survival analysis, ATX was an independent variable in patients with PBC. Increased ATX levels were associated with an approximately 4-fold higher risk of death/liver transplantation in patients with PBC and with a 2.6-fold higher risk in patients with PSC. This observation suggests that ATX may represent a valuable prognostic marker of survival in patients with chronic cholestatic conditions.

ATX mRNA levels were previously measured in various tissues and organs, including the brain, adipose tissue, lung, liver, intestine, kidney, ovary and high endothelial venules. However, the source of the circulating protein under normal conditions has not been identified^19^. A recent study indicated that intestinal enteroendocrine cells may be an important source of serum ATX activity in men with cholestasis^20^. Moreover, the *in vitro* expression of ATX can be induced by cytokines such as TNF activating the NF-kb pathway^21^. In addition to enhanced expression, increased levels of the enzyme might be the result of reduced elimination from the circulation. In mice, radioactive-labelled ATX was shown to be largely cleared from the circulation by the liver with a half-life time of a few minutes. It was suggested that ATX was cleared from plasma by scavenger receptors on liver sinusoidal endothelial cells, which are also involved in liver fibrosis^22^. In contrast, ATX was also increased in hepatectomized rats^21^. An effect of renal dysfunction on circulating ATX levels is unlikely, as serum creatinine was not correlated with ATX levels in our cohorts.

ATX expression was also found to be augmented in various malignancies such as lymphoma, malignant melanoma and breast cancer^23^. As LPA promotes tumour development and progression, it was speculated that increased ATX expression might lead to hepatocellular carcinoma^24^. However, it has recently been shown that ATX expression was comparable in hepatocellular carcinoma tissue and peri-tumorous fibrotic tissue and the ATX levels in the systemic circulation did not change after radiofrequency ablation of the tumour^25^. Due to the very low incidence of hepatocellular carcinoma in PBC and PSC patients, we were unable to analyse the effect of HCC on ATX levels in our cohorts.

Increased ATX in serum has also been found in patients with liver diseases such as chronic hepatitis C infection^26^ and HCV-associated fibrosis and cirrhosis^8^,^27^. Our study expands these observations by demonstrating that circulating ATX levels are increased in both non-cirrhotic and cirrhotic patients with chronic cholestasis. This finding is particularly relevant in patients with intrahepatic cholestasis of pregnancy in whom chronic liver damage is absent but ATX levels are highly increased^12^.

The potential pruritogenic role of ATX can be argued. Pruritogen should be secreted into bile as an interruption of enterohepatic circulation by nasobiliary drainage, but ATX is not present in bile^1^. Moreover, elevated ATX levels have been observed in other conditions unrelated to cholestatic itch such as regular pregnancy^12^. Therefore, it is possible that ATX can be produced in response to not-yet-identified biliary factors^3^ and potentiate rather than initiate the itch sensation through its product, LPA^5^. Amongst potential biliary suspects, bile acids were favoured; however, no correlation has been shown between itch severity and serum or skin level of any of the natural bile salts in any experimental or clinical studies^28^. We found that total bile acids concentration as well as several bile acids species were significantly correlated with ATX activity. Prominent correlations were obtained with regard to CDCA and CA, the natural farnesoid X receptor (FXR) agonists. Obeticholic acid, a semi-synthetic analogue of CDCA that selectively activates FXR in high dosages, increased ATX levels (unpublished) and caused pruritus^29^. Interestingly, a recent *in vitro* study demonstrated that natural bile salts act as partial non-competitive inhibitors of ATX, providing a molecular basis for the association between lysophosphatidic acid signalling and cholestatic disorders^30^.

Our study has some limitations. The data were derived from a clinical study, which does not allow us to define the exact molecular mechanisms behind the regulation of ATX expression in cholestatic patients. Moreover, the study pertains to Polish individuals, and our findings certainly require validation in further cohorts. Our findings are focused on short-term and general (but not liver-related) survival. Finally, the quality-of-life tools applied are not specifically designed for use in patients with PSC.

In conclusion, our study provides novel clinical evidence of the pruritogenic role of the ATX/LPA axis in the pathogenesis of cholestatic itch. The use of validated HRQoL questionnaires gave us the unique opportunity to detect the major associations between elevated ATX activity and other cholestasis-related symptoms such as fatigue and cognitive and emotional decline. ATX inhibitors or LPA receptor blockers may therefore be of use as therapeutic agents in the treatment of pruritus as well as other incapacitating symptoms related to chronic cholestasis. Moreover, ATX activity is correlated with the severity of cholestatic liver disease measured both by clinical and laboratory parameters as well as Mayo Risk scores. Finally, ATX is a significant predictor of death and liver transplantation. These findings do not undermine the key role of existing clinical parameters but suggest that ATX may represent an additional valuable indicator of hepatic injury and a novel prognostic marker in chronic cholestatic conditions.

## Methods

### Patients

In this cross-sectional study, we included two independent prospective cohorts of Caucasian patients diagnosed with PBC and PSC. These individuals had been recruited at two centres: the Liver Unit, Pomeranian Medical University in Szczecin, and the Liver and Internal Medicine Unit, Department of General, Transplant and Liver Surgery of the Medical University of Warsaw, from September 2009 to October 2014. Both diseases had been diagnosed according to EASL criteria^31^. Additionally, blood samples from 109 healthy Caucasian subjects (males/females: 51/58, mean age: 42.6 ± 11.5 years) collected at the University of Erlangen-Nürnberg served as controls for serum ATX activity and total bile acid concentration. Written informed consent was obtained from each subject included in the study. The study protocol was approved by the Ethics Committee of Pomeranian Medical University and conforms to the ethical guidelines of the 1975 Declaration of Helsinki (6^th^ revision, 2008). The study was performed according to the STROBE (Strengthening the Reporting of Observational Studies in Epidemiology) statement.

#### PBC group

One hundred and eighteen patients with PBC (males/females: 9/109; mean age: 57.7 ± 11.4 years) were studied. Fifty-three (46.1%) subjects with PBC had liver cirrhosis confirmed with either histology or imaging techniques (computed tomography or abdominal ultrasound). As shown in Table 4, patients with PBC had significantly elevated cholestatic parameters and moderately increased transaminases. Among the cirrhotic patients, 29 (54.7%) subjects were in the Child-Pugh class A group, 18 (34.0%) were in in the Child-Pugh class B group and 6 (11.3%) were in the Child-Pugh class C group. Fifty-three (49.5%) patients with PBC were UDCA non-responders according to the Barcelona definition^32^ (UDCA response data were available in 107 patients). UDCA non-response was associated with the presence of cirrhosis (chi^2^ = 8.5, *P = *0.004), Child-Pugh score (7.2 ± 1.6 points in non-responders *vs*. 6.1 ± 1.4 points in responders, *P = *0.02) and Mayo Risk Score (6.3 ± 1.5 points in non-responders *vs*. 5.5 ± 2.0 points in responders, *P = *0.03). A weak relationship was also detected between UDCA responsiveness and Child-Pugh class (chi^2^ = 5.7, *P = *0.05). UDCA non-responders did not differ from UDCA responders with respect to gender or MELD score (data not shown).

#### PSC group

One hundred and fifteen patients with PSC (males/females: 75/40; mean age: 35.4 ± 13.3 years) were studied. A diagnosis of IgG4-related cholangitis was excluded based on serum IgG4 levels. Patients with PSC were, as expected, younger than subjects with PBC and included more males. Thirty (26.1%) patients with PSC had also been diagnosed with cirrhosis: 16 (53.3%) were in the Child-Pugh class A group, 12 (40%) were in the Child-Pugh class B group, and 2 (6.7%) were in the Child-Pugh class C group. Parameters of cholestasis were elevated, and MELD scores and Mayo Risk scores in patients with PSC were low. These data are summarized in Table 4.

The number of patients treated at our institutions during the study period determined the size of the groups. Potential confounders or effect modifiers have, to the best of our knowledge, been excluded: none of the included subjects suffered from other conditions that could significantly influence HRQoL such as decompensated diabetes mellitus, renal insufficiency requiring dialyses, malignancy, heart failure ≥ NYHA II, rheumatoid arthritis or asthma. Clinical data, Mayo Risk score for PBC and PSC, and responses to HRQoL questionnaires were collected when the patients agreed to participate in the study. Missing data are indicated if present. Liver biochemistry, ATX activity and protein levels and bile acids concentrations were analysed in serum from subjects after fasting. Samples pertaining to the two cholestatic groups were analysed together in the same batch by laboratory personnel blinded to the clinical data.

### Quantification of serum ATX activity

Blood samples were allowed to clot before they were centrifuged at 4 °C, and serum was cryopreserved in aliquots at −80 °C. ATX activity was quantified in diluted sera by an examiner blinded to the clinical and serological groups. Serum samples were incubated with a buffer containing 500 mmol/L of NaCl, 5 mmol/L of MgCl_2_, 100 mmol/L of Tris (pH = 9.0), and 0.05% Triton X-100 for 60 minutes at 37 °C. Parallel incubations were performed in the presence and absence of 1 mmol/L of lysophosphatidylcholine (LPC) (14:0). The lysophospholipase activity of ATX was determined by measuring the amount of liberated choline as detected by enzymatic fluorimetry using choline oxidase (2 U/mL), horseradish peroxidase (1.6 U/mL), and homovanillic acid as substrates for peroxidase. After the addition of both enzymes to a buffer (consisting of 20 mmol/L of CaCl_2_, 2 mmol/L of HVA, 50 mmol/L of 3-[N-morpholino]propanesulfonic acid [pH = 8.0], and 0.1% Triton X-100), the increase in fluorescence was monitored at 37 °C on a NOVOstar analyser (excitation 320 nm and emission 405 nm; BMG Labtech GmbH, Offenburg, Germany). The (endogenous) amount of choline present in the sample without the addition of LPC was subtracted from the amount measured in the presence of LPC. Inter-assay variance was less than 15%, and intra-assay variance was below 10%.

### ATX protein quantification

ATX protein content in serum samples was quantified using an in-house optimized sandwich enzyme-linked immunosorbent assay specific for ATX. Briefly, microtiter plates were coated with an anti-human ATX antibody (R&D Systems, Minneapolis, MN, USA). Samples and recombinant ATX standards (R&D Systems, Minneapolis, MN, USA) were diluted in phosphate-buffered saline containing bovine serum albumin and 0.05% Tween-20. Captured antigen was detected with biotinylated anti-human ATX antibody (R&D Systems, Minneapolis, MN, USA) and streptavidin-horseradish peroxidase using tetramethylbenzidine as the chromogenic substrate. The presence of bilirubin and bile salts in cholestatic samples did not interfere with the quantification of ATX.

### Bile acids determination

Blood samples for bile acids determination were collected in EDTA-coated tubes. Immediately after collection, plasma was purified through centrifugation at 4 °C. Subsequently, one volume of formic acid 0.5 M was added to plasma samples. Samples were then frozen and kept at −80 °C until analyses. Further analyses were performed as described in detail elsewhere^33^,^34^,^35^,^36^.

### Assessment of health-related quality of life (HRQoL)

The HRQoL was assessed with both generic (The Medical Outcomes Study Short Form-36, SF-36) and disease-specific questionnaires (PBC-40 and PBC-27)^37^. The PBC-40 questionnaire contains 40 questions in the following domains: *Fatigue*, *Cognitive*, *Social-Emotional*, *Itch* and *Other Symptoms*, with higher scores indicating a worse HRQoL. PBC-27 is a simplified questionnaire for PBC^38^. SF-36 is a widely used and validated HRQoL questionnaire that includes 36 items divided into 8 scales. Scores can be obtained for each scale or can be aggregated into 2 summary scores: a *Mental Component Summary* score and a *Physical Component Summary* score. Scores range from between 0 and 100, with higher scores indicating a better HRQoL^39^.

### Statistics

The data are presented as mean ± standard deviation (SD) for continuous variables. The data were analysed using Stat-View-5 Software (SAS Institute, Cary, NC, US) and SPSS 20.0 (IBM Ehnigen, Germany) and included Fisher’s exact test and ANOVA when appropriate. Categorical data were compared using Levene’s test for equality of variances, and both pooled-variance and separate-variance t-tests were performed to assess the equality of the mean values. Correlation analyses were performed using the Spearman rank or Pearson’s correlation methods. Survival analysis was performed using the Cox regression model. Death and liver transplantation were considered events. Follow-up time was time until death, liver transplantation or the last contact with the patient. Serum ATX cut-off levels predictive of liver cirrhosis and overall survival in PBC and PSC patients were determined using the receiver operating characteristic (ROC) area under the curve (AUC) analysis. Associations between ATX levels and overall survival hazard ratios (HR) were calculated. To identify independent relationships and adjust the effects of covariates, multiple linear regression analyses were performed including all parameters with significant correlations (*P* < 0.05) in the univariate analysis as covariates with regard to laboratory parameters, prognostic scales and HRQoL. Multivariate survival analysis was performed using Cox’s regression model. A *P* value of <0.05 was considered statistically significant.

## Additional Information

**How to cite this article**: Wunsch, E. *et al*. Serum Autotaxin is a Marker of the Severity of Liver Injury and Overall Survival in Patients with Cholestatic Liver Diseases. *Sci. Rep*. **6**, 30847; doi: 10.1038/srep30847 (2016).

## Supplementary Material

Supplementary Information

## Figures and Tables

**Table 1:** Associations between ATX and laboratory data (Pearson’s correlation coefficients).

Feature	PBC (n = 118)	PSC (n = 115)
**Haemoglobin** (g/dL)	**r = −0**.**362** (***P***** < 0**.**001**)	**r = −0**.**289** (***P***** < 0**.**01**)
**Platelets** (10^3/μL, Normal: 150–400)	r = −0.133 (*P = *0.17)	**r = −0**.**328** (***P***** < 0**.**001**)
**ALT** (IU/L; Normal: <30)	r = 0.009 (*P = *0.93)	r = 0.063 (*P* = 0.51)
**AST** (IU/L; Normal: <30)	r = 0.139 (*P = *0.15)	**r = 0.352 (*****P***** < 0.001)**
**ALP** (IU/L; Normal: <120)	**r = 0**.**336** (***P***** < 0**.**001**)	**r = 0**.**362** (***P***** < 0**.**0001**)
**GGT** (IU/L; Normal: <42)	r = −0.002 (*P = *0.99)	r = −0.032 (*P = *0.74)
**Bilirubin** (mg/dL; Normal <1.0)	r = 0.083 (*P = *0.39)	r = 0.155 (*P = *0.11)
**Albumin** (g/dL; Normal: 3.8–4.4)	**r = −0**.**263** (***P***** < 0**.**01**)	**r = −0**.**325** (***P***** < 0**.**001**)
**INR** (Normal: 0.8–1.2)	r = 0.119 (*P = *0.22)	**r = 0**.**198** (***P = *****0**.**04**)
**Total bile acids** (nM)	**r = 0**.**417** (***P***** < 0**.**0001**)	**r = 0**.**409** (***P***** < 0**.**0001**)
**Creatinine** (mg/dL; Normal: <1.0)	r = 0.002 (*P = *0.99)	r = −0.084 (*P = *0.39)

**Table 2:** Correlation between ATX and bile acid profiles in analysed groups.

Bile acid species	PBC group (n = 118)		PSC group (n = 108)	
	Correlation coefficient (r)	*P* value	Correlation coefficient (r)	*P* value
CDCA	0.084	0.37	−0.109	0.26
GCDCA	**0**.**360**	**<0**.**0001**	**0**.**462**	**<0**.**0001**
TCDCA	**0**.**363**	**<0**.**0001**	**0**.**412**	**<0**.**0001**
CA	0.01	0.90	−0.008	0.94
GCA	**0**.**417**	**<0**.**0001**	**0**.**381**	**<0**.**0001**
TCA	**0**.**365**	**<0**.**0001**	**0**.**308**	**<0**.**01**
DCA	**−0**.**243**	**<0**.**01**	**−0**.**198**	**0**.**04**
GDCA	0.048	0.61	0.150	0.12
TDCA	**0**.**237**	**0**.**01**	0.138	0.16
LCA	0.103	0.27	0.134	0.17
GLCA	0.172	0.06	0.182	0.06
TLCA	**0**.**338**	**<0**.**001**	**0**.**221**	**0**.**02**
LCA-S	0.109	0.25	**0**.**233**	**0**.**02**
HCA	0.132	0.16	**0**.**252**	**<0**.**01**
HDCA	**−0**.**192**	**0**.**04**	−0.063	0.52
UDCA	**0**.**214**	**0**.**02**	0.01	0.91
GUDCA	**0**.**278**	**<0**.**01**	**0**.**308**	**<0**.**01**
TUDCA	**0**.**318**	**<0**.**001**	**0**.**396**	**<0**.**0001**
Total bile acids	**0**.**412**	**<0**.**0001**	**0**.**409**	**<0**.**0001**

**Table 3:** Correlations between serum ATX and health-related quality of life.

	PBC group*		PSC group**	
	r	*P* value	r	*P* value
**PBC-40**				
*Other symptoms*	r = 0.135	*P = *0.8	r = 0.02	*P = *0.83
*Itch*	**r = 0**.**305**	***P***** < 0**.**01**	**r = 0**.**376**	***P***** < 0**.**0001**
*Fatigue*	**r = 0**.**218**	***P = *****0**.**02**	r = 0.062	*P = *0.51
*Cognitive*	**r = 0**.**195**	***P = *****0**.**04**	r = 0.019	*P = *0.84
*Social and Emotional*	r = 0.175	*P = *0.06	r = −0.067	*P = *0.48
**PBC-27**				
*Other symptoms*	r = 0.071	*P = *0.45	r = 0.045	*P = *0.63
*Dryness*	r = 0.087	*P = *0.36	r = 0.001	*P = *0.99
*Itch*	**r = 0**.**305**	***P***** < 0**.**01**	**r = 0**.**376**	***P***** < 0**.**0001**
*Fatigue*	**r = 0**.**217**	***P = *****0**.**02**	r = 0.039	*P = *0.68
*Cognitive*	**r = 0**.**207**	***P = *****0**.**03**	r = 0.016	*P = *0.9
*Emotional*	**r = 0**.**202**	***P = *****0**.**03**	r = −0.092	*P = *0.33
*Social*	r = 0.086	*P = *0.37	r = −0.018	*P = *0.85
**SF-36**				
*Physical Functioning*	**r = −0**.**204**	***P = *****0**.**03**	r = −0.103	*P = *0.29
*Role Physical*	r = −0.146	*P = *0.13	r = 0.057	*P = *0.55
*Bodily Pain*	r = −0.037	*P = *0.7	r = 0.073	*P = *0.44
*General Health*	r = −0.108	*P = *0.27	r = 0.056	*P = *0.56
*Vitality*	r = −0.158	*P = *0.1	r = 0.04	*P = *0.67
*Social Functioning*	r = −0.142	*P = *0.14	r = 0.042	*P = *0.66
*Role Emotional*	r = −0.07	*P = *0.48	r = 0.02	*P = *0.83
*Mental Health*	r = −0.167	*P = *0.08	r = 0.128	*P = *0.18
*Physical Component Summary*	r = −0.141	*P = *0.15	r = 0.067	*P = *0.48
*Mental Component Summary*	r = −0.147	*P = *0.13	r = 0.053	*P = *0.58

^*^PBC group: for PBC-40/PBC-27, data available for 113 patients; for SF-36 data available for 106 patients.

^**^PSC group: for PBC-40/PBC-27, data available for all patients (n = 115); for SF-36 data available for 113 patients.

**Table 4:** Clinical and laboratory characteristics of analysed patients.

Feature	PBC (n = 118)	PSC (n = 115)	*P* value
**Age** (years)	57.7 ± 11.4	35.4 ± 13.3	**<0**.**0001**
**Gender** (M/F)	9 (7.6%)/109 (92.4%)	75 (65.2%)/40 (34.8%)	**<0**.**0001**
Cirrhosis* (yes/no)	53 (46.1%)/62 (53.9%)	30 (26.1%)/85 (73.9%)	**<0**.**01**
Child-Pugh class** (A/B/C)	29 (54.7%)/18 (34.0%)/6 (11.3%)	16 (53.3%)/12 (40%)/2 (6.7%)	0.73
**Haemoglobin** (g/dL)	12.4 ± 1.7	13.1 ± 2.0	**0**.**01**
**Platelets** (10^3/μL, Normal: 150–400)	190 ± 117	227 ± 110	**0**.**02**
**ALT** (IU/L; Normal: <30)	80 ± 114	93 ± 90	0.34
**AST** (IU/L; Normal: <30)	77 ± 67	71 ± 51	0.48
**ALP** (IU/L; Normal: <120)	255 ± 206	299 ± 195	0.11
**GGT** (IU/L; Normal: <42)	302 ± 506	279 ± 315	0.69
**Bilirubin** (mg/dL; Normal: <1.0)	2.6 ± 4.5	3.0 ± 5.6	0.56
**Albumin** (g/dL; Normal: 3.8–4.4)	3.8 ± 0.5	4.2 ± 0.5	**<0**.**0001**
**INR** (Normal: 0.8–1.2)	1.1 ± 0.2	1.1 ± 0.1	0.36
**Total bile acids** (nM)	43 850.1 ± 47081.1	44 751.7 ± 58 058.1	0.79
**Creatinine** (mg/dl; Normal: <1.0)	0.9 ± 0.4	0.8 ± 0.2	**<0**.**001**
**UDCA response** (yes/no)***	54 (50.5%)/53 (49.5%)	—	—
Mayo Risk Score****	5.9 ± 1.8	−0.1 ± 1.3	—
**MELD**	9.7 ± 4.2	9.8 ± 4.5	0.77
**ATX activity** (nmol/mL*min)	10.2 ± 4.4	7.3 ± 3.4	**<0**.**0001**

^*^Cirrhosis: for the PBC group, no data for 3 patients.

^**^Child-Pugh classes calculated only for cirrhotic patients.

^***^UDCA response data available for 107 patients.

^****^Calculated separately for PBC and PSC.
