# Supplementary material for: Serum Autotaxin is a Marker of the Severity of Liver Injury and Overall Survival in Patients with Cholestatic Liver Diseases
Source: Sci Rep. 2016 Aug 10;6:30847. doi: 10.1038/srep30847 (PMC4978954; doi:10.1038/srep30847)
Supplement: Supplementary Information [file srep30847-s1.doc]

**SUPPLEMENTARY MATERIALS**

**Manuscript Title: Serum Autotaxin is a Marker of the Severity of Liver Injury and Overall Survival in Patients with Cholestatic Liver Diseases.**

**Authors**: Ewa Wunsch, Marcin Krawczyk, Malgorzata Milkiewicz, Jocelyn Trottier, Olivier Barbier, Markus F. Neurath, Frank Lammert, Andreas E. Kremer and Piotr Milkiewicz

**Supplementary Table 1. Associations between ATX and liver injury parameters in cirrhotic and non-cirrhotic patients (Pearson’s correlation coefficients).**

A. Patients with PBC

| **Feature** | **Cirrhosis (n = 53)** | **No cirrhosis (n = 62)** |
| --- | --- | --- |
| **Hemoglobin** (g/dl) | r = -0.124 (*P* = 0.4) | **r = -0.383 (*P =* 0.003)** |
| **Platelets** (10^3/uL, Normal: 150-400) | r = 0.193 (*P* = 0.19) | r = -0.036 (*P =* 0.79) |
| **ALT** (IU/l; Normal:<30) | r = 0.263 (*P* = 0.06) | r = 0.046 (*P* = 0.73) |
| **AST** (IU/l; Normal:<30) | r = 0.163 (*P* = 0.26) | r = 0.110 (*P =* 0.41) |
| **ALP** (IU/l; Normal:< 120) | **r = 0.34 (*P =* 0.01)** | **r = 0.313 (*P* = 0.01)** |
| **GGT** (IU/l; Normal:< 42) | r = 0.054 (*P* = 0.71) | r = 0.055 (*P* = 0.69) |
| **Bilirubin** (mg/dl; Normal <1.0) | r = 0.087 (*P* = 0.54) | r = -0.015 (*P* = 0.91) |
| **Albumin** (g/dl; Normal: 3.8 - 4.4) | r = 0.076 (*P =* 0.62) | **r = -0.277 (*P =* 0.04)** |
| **INR** (Normal: 0.8-1.2) | r = 0.068 (*P* = 0.64) | r = - 0.046 (*P* = 0.74) |
| **Total bile acids** (nM) | r = 0.229 (*P* = 0.1) | **r = 0.492 (*P* < 0.0001)** |
| **Mayo Risk Score for PBC** (points) | r = -0.019 (*P* = 0.9) | **r = 0.313 (*P* = 0.02)** |
| **MELD score** (points) | r = 0.061 (*P* = 0.68) | r = 0.136 (P = 0.33) |

**B. Patients with PSC**

| **Feature** | **Cirrhosis (n = 30)** | **No cirrhosis (n = 85)** |
| --- | --- | --- |
| **Hemoglobin** (g/dl) | r = -0.017 (*P* = 0.94) | **r = -0.345 (*P =* 0.018)** |
| **Platelets** (10^3/uL, Normal: 150-400) | r = 0.180 (*P* = 0.34) | **r = -0.340 (*P =* 0.002)** |
| **ALT** (IU/l; Normal:<30) | r = -0.208 (*P* = 0.27) | r = 0.210 (*P* = 0.06) |
| **AST** (IU/l; Normal:<30) | r = 0.060 (*P* = 0.75) | **r = 0.376 (*P =* 0.005)** |
| **ALP** (IU/l; Normal:< 120) | r = 0.323 (*P =* 0.08) | **r = 0.342 (*P* = 0.015)** |
| **GGT** (IU/l; Normal:< 42) | r = -0.209 (*P* = 0.27) | r = 0.040 (*P* = 0.73) |
| **Bilirubin** (mg/dl; Normal <1.0) | r = 0.074 (*P* = 0.71) | r = 0.049 (*P* = 0.67) |
| **Albumin** (g/dl; Normal: 3.8 - 4.4) | r = -0.204 (*P =* 0.28) | r = -0.217 (*P* = 0.06) |
| **INR** (Normal: 0.8-1.2) | r = -0.124 (*P* = 0.52) | r = 0.160 (*P* = 0.16) |
| **Total bile acids** (nM) | r = 0.073 (*P* = 0.71) | **r = 0.481 (*P* < 0.0001)** |
| **Mayo Risk Score for PBC** (points) | r = 0.253 (*P* = 0.18) | **r = 0.321 (*P* = 0.005)** |
| **MELD score** (points) | r = 0.096 (*P* = 0.62) | **r = 0.213 (*P* = 0.06)** |

**Supplementary Table 2.** Measures of health-related quality of life in analysed patients.

|  | **PBC group*** | **PSC group**** | ***P* value** |
| --- | --- | --- | --- |
| **PBC-40** | | | |
| *Other symptoms* | 17.0 ± 5.2 | 12.9 ± 4.5 | **< 0.0001** |
| *Itch* | 5.9 ± 4.8 | 4.4 ± 4.0 | **0.01** |
| *Fatigue* | 29.6 ± 10.7 | 25.0 ± 10.0 | **< 0.001** |
| *Cognitive* | 14.1 ± 5.5 | 11.1 ± 5.1 | **< 0.0001** |
| *Social and Emotional* | 32.9 ± 10.5 | 29.7 ± 10.8 | **0.02** |
| **PBC-27** | | | |
| *Other symptoms* | 8.3 ± 2.8 | 6.1 ± 2.5 | **< 0.0001** |
| *Dryness* | 5.7 ± 2.3 | 4.0 ± 1.8 | **< 0.0001** |
| *Itch* | 5.9 ± 4.8 | 4.4 ± 4.0 | **0.01** |
| *Fatigue* | 21.7 ± 7.3 | 18.6 ± 7.2 | **< 0.01** |
| *Cognitive* | 11.7 ± 4.6 | 9.3 ± 4.3 | **< 0.0001** |
| *Emotional* | 7.7 ± 3.2 | 6.9 ± 3.1 | 0.05 |
| *Social* | 7.9 ± 3.0 | 6.8 ± 3.3 | **0.01** |
| **SF-36** | |  |  |
| *Physical Functioning* | 57.0 ± 26.7 | 83.0 ± 20.8 | **< 0.0001** |
| *Role Physical* | 32.9 ± 38.5 | 64.7 ± 40.9 | **< 0.0001** |
| *Bodily Pain* | 55.2 ± 28.2 | 70.9 ± 28.6 | **< 0.0001** |
| *General Health* | 43.5 ± 17.4 | 48.4 ± 22.3 | 0.07 |
| *Vitality* | 46.4 ± 21.0 | 54.4 ± 18.8 | **< 0.01** |
| *Social Functioning* | 61.1 ± 24.5 | 66.1 ± 24.7 | 0.18 |
| *Role Emotional* | 54.8 ± 45.8 | 76.0 ± 36.5 | **< 0.001** |
| *Mental Health* | 61.4 ± 20.3 | 65.1 ± 19.8 | 0.18 |
| *Physical Component Summary* | 46.9 ± 22.4 | 64.7 ± 21.4 | **< 0.0001** |
| *Mental Component Summary* | 56.0 ± 23.1 | 62.3 ± 20.9 | **0.03** |

* PBC group: for PBC-40/PBC-27 data available for 115 patients, for SF-36 data available for 109 patients

** PSC group: for PBC-40/PBC-27 data available for all patients (n=115), for SF-36 data available for 113 patients

**Supplementary Table 3.** Multivariate survival analyses (Cox's regression model).

A. Patients with PBC

| **Variables in the Equation**   |  | | | | | | | | | | --- | --- | --- | --- | --- | --- | --- | --- | --- | | Walddf*P* valueHR95.0% CI for HR | | | | | | | | | | BSE |  |  |  |  |  |  | Lower | | | 0.020 | 0.559 | | Presence of cirrhosis  MELD | 0.846  0.128 | 0.585  0.055 | 2.087  5.423 | 1  1 | 0.149  **0.020** | 2.330  1.137 | 0.740  1.021 | 7.337  1.267 | | ATX cut-off | 1.064 | 0.528 | 4.063 | 1 | **0.044** | 2.897 | 1.030 | 8.150 | | Mayo Risk score for PBC | 0.056 | 0.089 | 0.396 | 1 | 0.529 | 1.057 | 0.889 | 1.259 | |  |  |  |  |  |  |  |  |  |   **B.** **Patients with PSC**  **Variables in the Equation** | | | | | | | | |
| --- | --- | --- | --- | --- | --- | --- | --- | --- |
|  | B | SE | Wald | df | *P* value | HR | 95.0% CI for HR | |
|  |  |  |  |  |  |  | Lower | Upper |
| Presence of cirrhosis | -0.136 | 0.446 | 0.094 | 1 | 0.760 | 0.872 | 0.364 | 2.091 |
| MELD  ATX cut-off | 0.199  0.297 | 0.038  0.442 | 26.869  0.452 | 1  1 | **0.000**  0.502 | 1.220  1.346 | 1.132  0.566 | 1.315  3.198 |

**Supplementary Figure 1.**


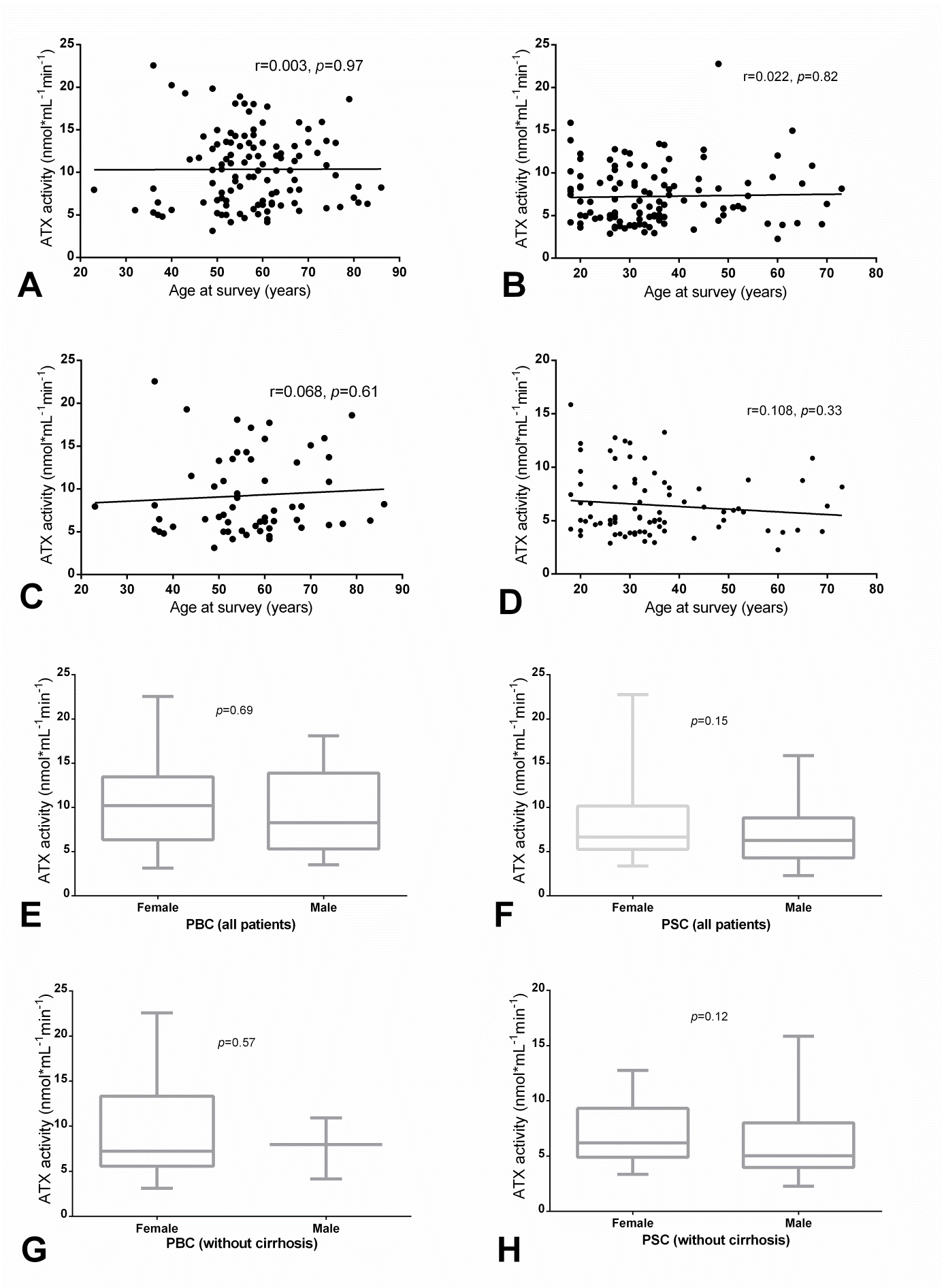


**Supplementary Figure 2.**

**
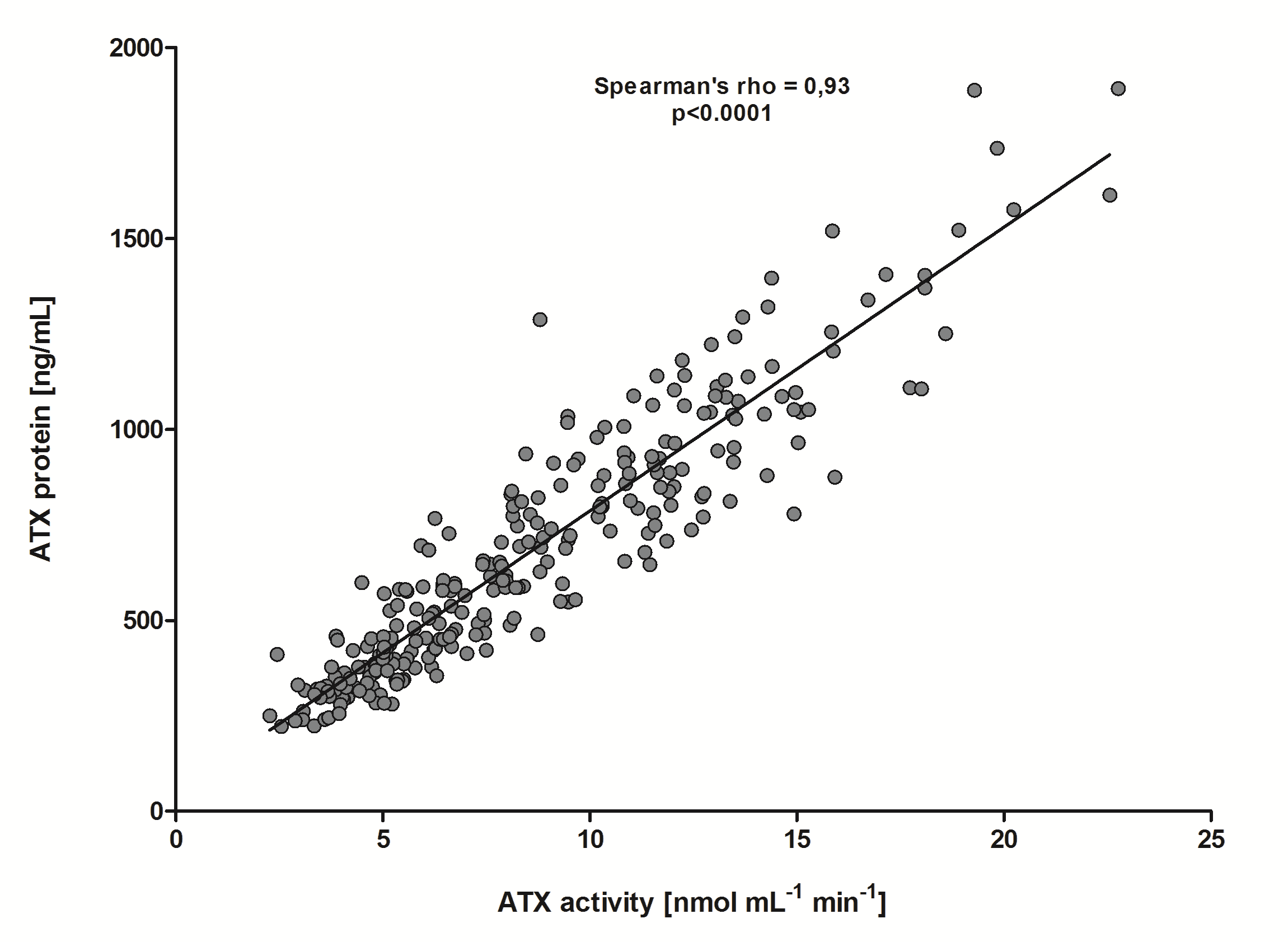
**

**Supplementary Figure 3.**

**
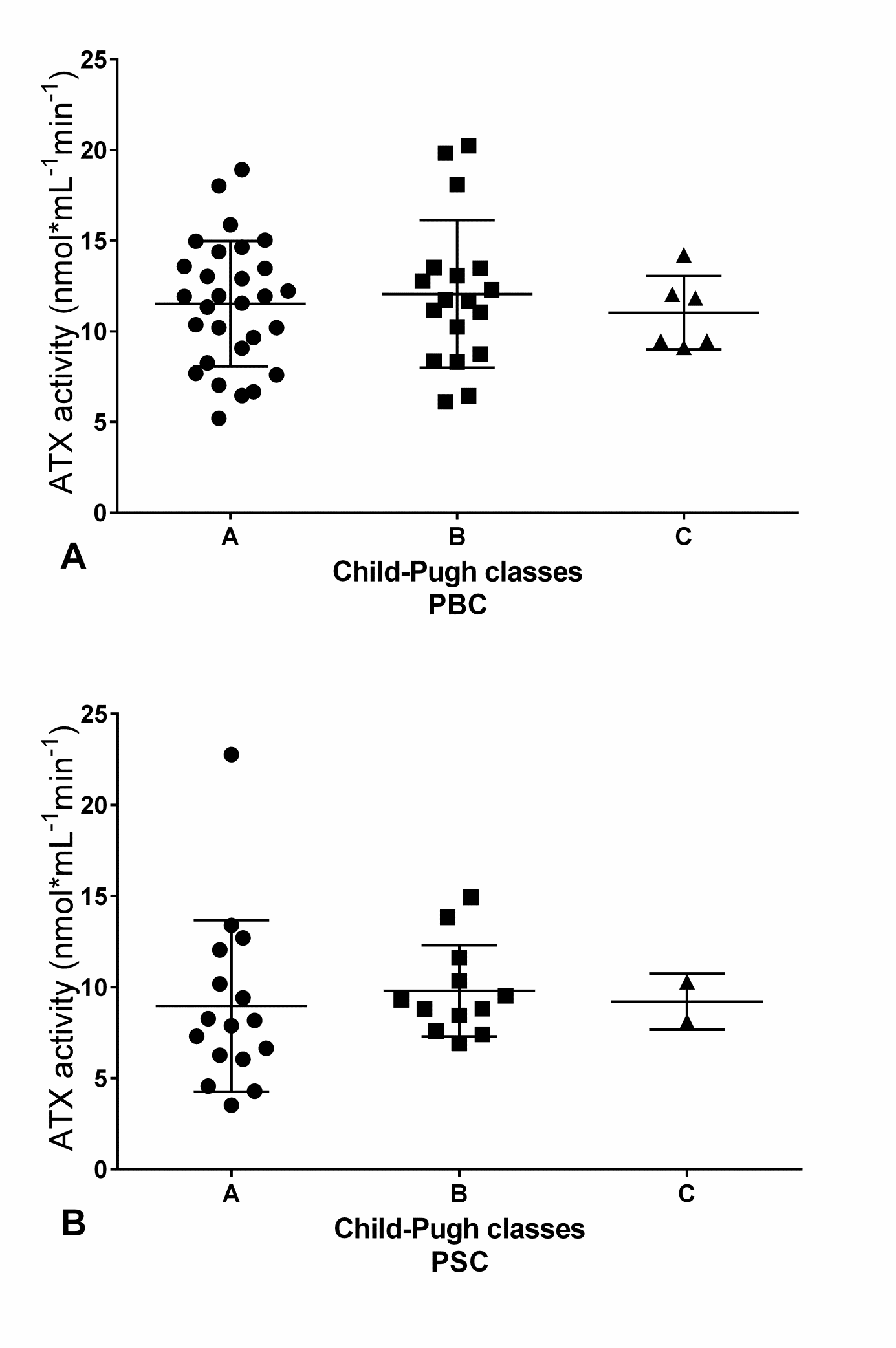
**

**Supplementary Figure 4.**

**
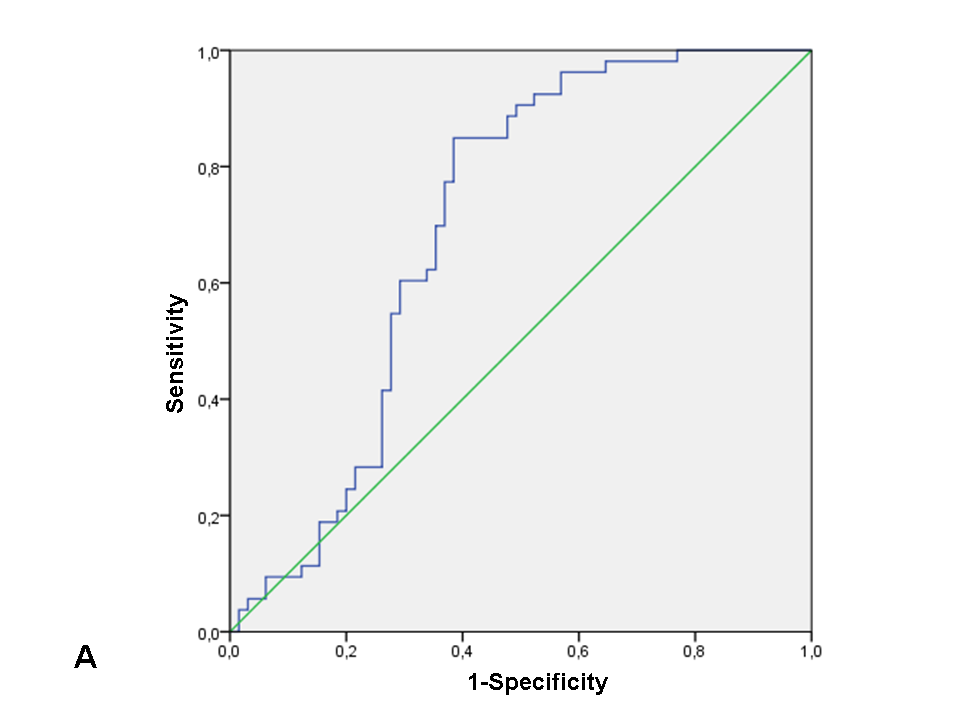
**

**
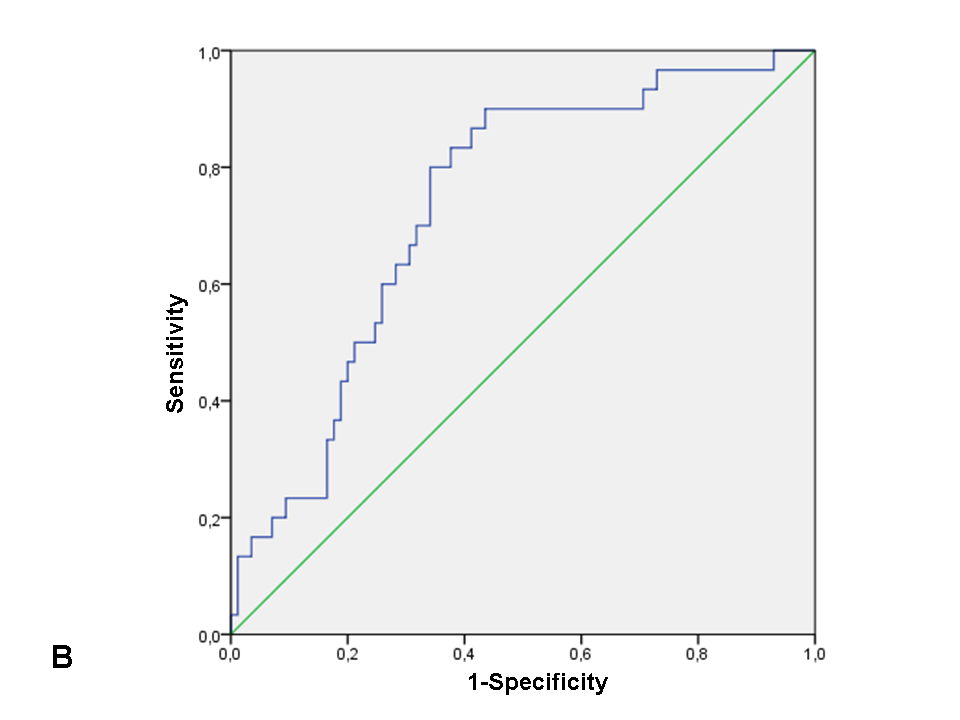
**

**Supplementary Figure 5.**

**
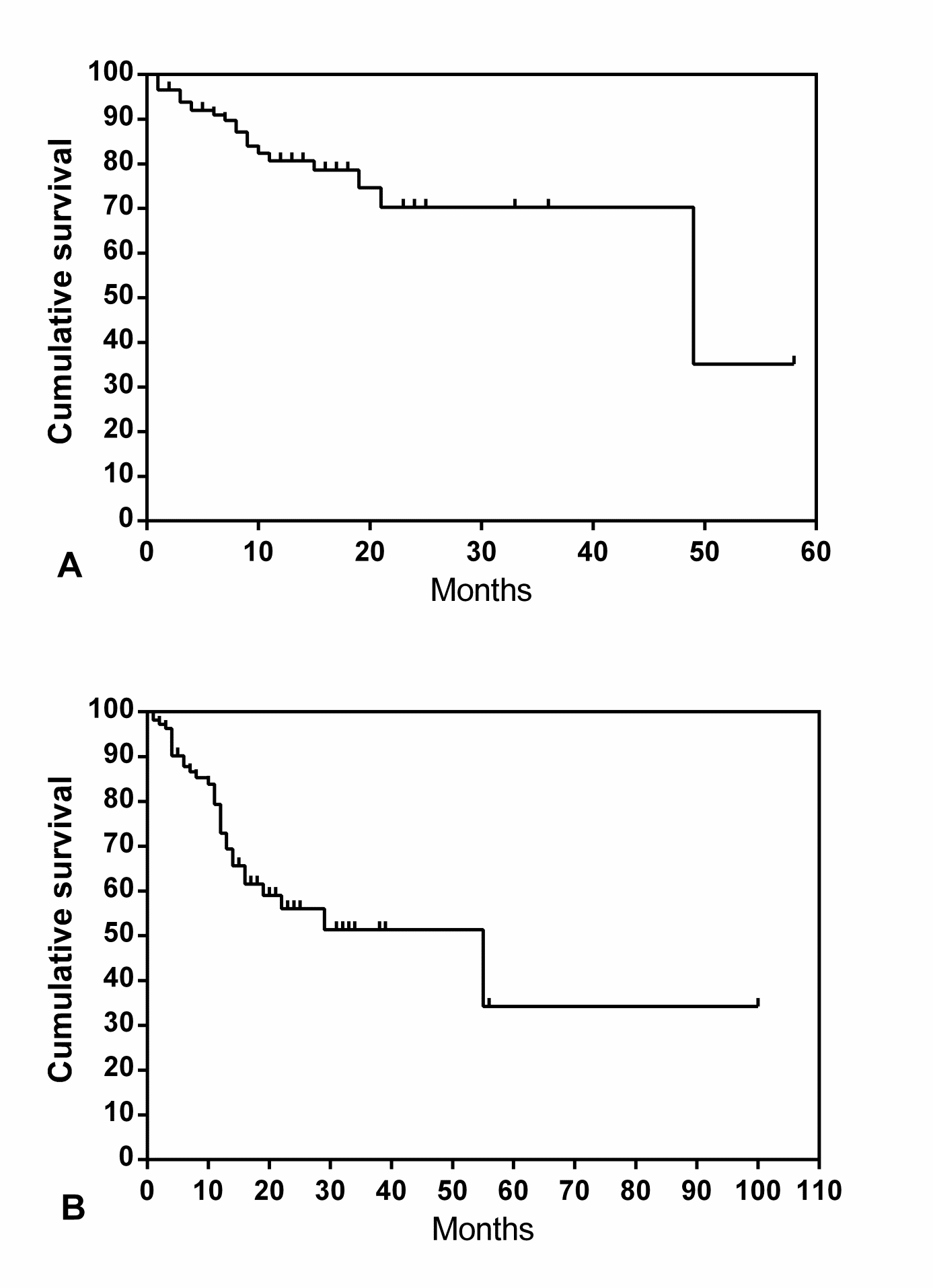
**

**Supplementary figures legends**

**Figure 1S. Relationship between ATX activity and age and gender of the included patients.** ATX activity was independent from patients’ age in both (A) PBC and (B) PSC group, even when patients with cirrhosis were excluded in both (C) PBC and (D) PSC group (Pearson’s correlation coefficients). Comparisons of ATX activity in all male and female patients with (E) PBC and (F) PSC as well as in non-cirrhotic male and female patients with (G) PBC and (H) PSC (ANOVA analysis. Data are presented as box-and-whisker plots with median value).

**Figure 2S. ATX activity closely correlates with ATX protein levels.** ATX activity showed a linear correlation with protein levels. Spearman’s correlation coefficient: r = 0.93, *P* < 0.0001.

**Figure 3S. Relationship between ATX activity and Child-Pugh classes in cirrhotic patients.** There were no differences in ATX level between cirrhotic patients in different Child-Pugh classes in both (A) PBC and (B) PSC group (ANOVA analysis. Data are presented as box-and-whisker plots with median value).

**Figure 4S. Receiver operating characteristic (ROC) area under the curve (AUC) analysis for prediction of liver cirrhosis (A) in patients with PBC and (B) with PSC.** The optimal cut-off of ATX activity for discriminating cirrhotics from non-cirrhotics was in PBC 9.56 nmol mL^-1^min^-1^ (with a sensitivity of 70%, specificity of 65%) and in PSC 7.43 nmol mL^-1^min^-1^ (with a sensitivity of 70%, specificity of 67%).

**Figure 5S. Cumulative survival in analysed patients.** Cumulative survival function in patients with (B) PBC and (B) PSC.
